# Supplementary material for: Effectiveness and Safety of Ayurvedic Medicines in Type 2 Diabetes Mellitus Management: A Systematic Review and Meta-Analysis
Source: Front Pharmacol. 2022 Jun 8;13:821810. doi: 10.3389/fphar.2022.821810 (PMC9213670; doi:10.3389/fphar.2022.821810)
Supplement: Supplementary file 1 [file DataSheet1.zip › Supplementary Material/Forest and Funnel Plots/Tinospora cordifolia/HDL-C.pdf]

Tinospora cordifolia - HDL-C

	Intervention			Comparator			Mean Diff.	Weight
Study	N	Mean	SD	N	Mean	SD	with 95% CI	(%)
Balasubramaniam 2010	20	24.00	5.00	20	24.00	6.00	0.00 [-3.42, 3.42]	73.22
Roy 2015	29	45.30	11.08	30	46.70	11.10	-1.40 [-7.06, 4.26]	26.78
**Overall**							-0.37 [-3.30, 2.55]	

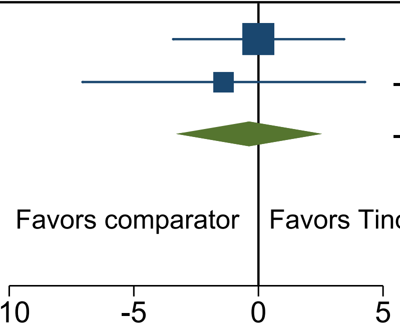

Heterogeneity: $\tau^2 = 0.00$, $I^2 = 0.00\%$, $H^2 = 1.00$

Test of $\theta_i = \theta_j$: $Q(1) = 0.17$, $p = 0.68$

Test of $\theta = 0$: $z = -0.25$, $p = 0.80$

Random-effects REML model
